# An online survey of users of tobacco vaporizers, reasons and modes of utilization, perceived advantages and perceived risks

**DOI:** 10.1186/s12889-019-6957-0

**Published:** 2019-05-27

**Authors:** Sébastien Queloz, Jean-François Etter

**Affiliations:** Institute of Global Health, Faculty of Medicine, University of Geneva, Geneva, Switzerland

**Keywords:** Tobacco vaporizer, E-cigarette, Nicotine, Tobacco withdrawal symptoms, Cigarette consumption, Internet

## Abstract

**Background:**

Tobacco vaporizers heat tobacco without burning it, to produce an inhalable aerosol. Various models have recently appeared on the market, mostly manufactured by the tobacco industry, but few of the studies published on tobacco vaporizers are independent from the manufacturers. The goals of this study were to explore who uses tobacco vaporizers, how these products are used, reasons for utilization, perceived advantages and risks.

**Methods:**

Online questionnaire collected from October 2016 to January 2018 in self-selected visitors aged > 18 to an anti-addiction website.

**Results:**

We obtained 170 valid responses, of whom 104 were using tobacco vaporizers. For homogeneity, we included only the 102 users of the Brand 1 tobacco vaporizer in our analysis, as there were only two users of other vaporizers.

Among these 102 vaporizer users, about half were current cigarette smokers (57%), the rest were former cigarette smokers. The median age was 41, and the median duration of utilization was 9 months.

Most (88%) used the vaporizer daily, 8% were occasional users and 4% were past users. Among current smokers, 80% were currently trying to reduce their cigarette consumption and 29% were trying to quit. The vaporizer was used mainly to replace cigarettes (94%), because it was perceived to be less toxic than cigarettes (89%), to help stop smoking or to avoid starting smoking again (72%), or to reduce cigarette consumption (71%).

Current smokers who were daily or occasional vaporizer users reported smoking a median of 8.0 cigarettes per day, compared with 20.0 per day before they started to use the vaporizer (*p* < .0001, Wilcoxon signed-rank test).

**Conclusions:**

In this online sample of early adopters, Brand 1 was by far the most frequently used tobacco vaporizer. It was used by current or former smokers only, mainly to replace cigarettes, and satisfaction ratings were good. Users considered the tobacco vaporizer to be less toxic than cigarette smoke and perceived it to be helpful for reducing or stopping smoking.

## Background

Tobacco vaporizers heat tobacco rather than burning it, to produce an inhalable aerosol [1]. They contain a battery-powered heating element and an insertion site for the tobacco refill sticks [1]. The vaporizers produce an inhalable aerosol that might be > 90% less toxic than the smoke produced by a combustible cigarette because of the temperature at which the tobacco is heated (around 300 °C instead of > 900 °C for a combustible cigarette) [2–4]. At this lower temperature pyrolysis occurs, but not combustion [5].

For more than 25 years, studies have tended to show that the aerosol produced by tobacco that is heated but not burned is less toxic than cigarette smoke [6–8]. Biomarker analysis in humans [9–11], animals [12, 13] and on human cultured cells [14, 15] also showed reduced toxicity from the aerosol produced by heated tobacco products compared to cigarette smoke. Nonetheless, the aerosol from tobacco vaporizers is not free of toxicologically active substances [5, 16–21], and some consider that such aerosols should be described as smoke because pyrolysis occurs in some devices [5].

Unlike e-cigarettes that heat liquids that can contain nicotine, tobacco vaporizers heat tobacco. The nicotine supplied by some vaporizers reaches the bloodstream at a speed approaching the delivery speed achieved by inhaling cigarette smoke [22], but at lower concentration [22, 23].

In recent years, different models of tobacco vaporizers have appeared on the market, mostly manufactured by the tobacco industry which is investing substantial sums of money in the research and development of these products. For instance, since November 2014 Philip Morris International (PMI) has sold a tobacco vaporizer named IQOS in various countries where it is commercially quite successful [1, 24], particularly in Japan where sales of IQOS refills represented 15.5% of the tobacco market in September 2018 [24]. In December 2016 PMI submitted an application to the United States Food and Drug Administration to register IQOS as a modified-risk tobacco product [1]. Similarly, British American Tobacco have launched a tobacco vaporizer named “glo” and invested one billion GBP in the development of new tobacco vaporizing devices [25].

Very few of the studies published on tobacco vaporizers are independent of the manufacturers [3, 5, 16, 19–21, 23, 26–28] and many questions remain unanswered. For example: who uses tobacco vaporizers, why and how are these products used, what are the perceived advantages and risks, what are the effects on cigarette consumption, are the vaporizers used by non-smokers, do they encourage cigarette consumption, are they addictive?

Independent research is needed to allow policy makers, legislators, clinicians, manufacturers, retailers and consumers to make informed decisions. Thus, the goals of this study were to explore who the tobacco vaporizers users are, how vaporizers are used, reasons for utilization, and perceived advantages and risks.

## Methods

### Qualitative phase

Participants were enrolled via the anti-addiction website www.stop-dependance.ch which is run by the second author. We conducted brief interviews by e-mail with eight self-selected visitors to this website who were aged 18 or older and were currently using, or had used, a tobacco vaporizer. We also conducted telephone interviews with five of these users to identify reasons for use, perceived advantages and drawbacks, opinions and satisfaction with the product. These qualitative data were used to design the closed-format questionnaire used in the quantitative phase.

### Quantitative phase

We posted a questionnaire in French and English on www.stop-dependance.ch and asked discussion forums, websites and anti-tobacco leagues to post a link to the questionnaire. We also posted this link on Facebook. Most respondents came from stop-dependance.ch.

Data were collected between October 2016 and January 2018.

Participants were aged 18 or older and they were using, or had used, a tobacco vaporizer (any brand). We recorded Internet Protocol (IP) addresses to identify and delete duplicate records.

Before answering the questionnaire, participants were informed that:

“Cigarette” referred to “real” combustible cigarette.

“E-cigarette” referred to a product that heats a liquid producing an aerosol that can be inhaled.

“Tobacco vaporizer” or “vaporizer” referred to a product that heats tobacco producing an aerosol that can be inhaled.

Only current or former users of tobacco vaporizers, using any brand or model, were included in the study.

The questionnaire covered:

- Current or past utilization of a tobacco vaporizer and intention to use it, brand and model (open-ended questions).
- Reasons for using one, duration and frequency of utilization, number of refills and puffs per day, number of puffs per refill (note: Brand 1 is designed to produce a maximum of 14 puffs per stick, and at the time of data collection only tobacco and menthol flavors were available for Brand 1 refill sticks [2]), monthly expenditure on vaporizers and refills.
- Assessment of the taste, perceived feelings, satisfaction and perceived advantages and disadvantages.
- Perceived risk and comparison of risk with combustible cigarettes.
- Current or past utilization of tobacco, age at smoking initiation, number of cigarettes per day, duration of smoking, time to first cigarette after waking [29] (combined to compute the Heaviness of Smoking Index, HSI [30],)
- Current or past utilization of nicotine replacement medications, other medication for stopping smoking or e-cigarettes. Intention to stop smoking or to reduce smoking, previous quit attempts, tobacco dependence (on a scale from 0 to 100) [31], confidence in ability to stop smoking
- Age, gender, country of residence
- Presence of a tobacco related disease, cannabis use and hazardous alcohol consumption (AUDIT-C) [32].

### Statistical analysis

Before starting this exploratory study, the intended sample size was 200, which would have allowed us to obtain a 95% confidence interval of +/− 7% for variables whose frequency is 50%, and of +/− 6% for a frequency of 25%. It would also have allowed us to detect a 20% difference between groups for dichotomic variables with a frequency of 50%, with a power of 80% and a *p* value of 0.05. We estimated that this level of precision was sufficient for this exploratory study.

We reported medians rather than means, because medians are less sensitive to extreme values. We compared current and former smokers using Mann–Whitney *U*-tests for medians, χ^2^ tests for proportions. Bonferroni correction was used for multiple comparisons and Wilcoxon signed-rank test was used to compare median cigarette consumption among current smokers before and after they started using a vaporizer. As we made 132 comparisons between current and former smokers, the corrected significance threshold was *p* = 0.05/132 = 0.0004. We indicated 95% confidence intervals for proportions in Tables 4 to 6. Prices in other currencies were converted to Euros.

#### Ethics and informed consent

The study protocol was submitted to the ethics committee of the canton of Geneva which did not examine it because the committee considered that this type of study (an online survey) did not require approval, according to the Swiss laws that regulate medical research.

We informed participants that their answers would be anonymously stored on a computer file for statistical analyses and that they would not be transmitted to third parties. We did not request a formal consent for participation, consent was implicit.

## Results

### Participation

#### Qualitative phase

Eight participants responded by e-mail, of whom five were also interviewed by telephone; two were former Brand 1 tobacco vaporizer users, and six were current Brand 1 vaporizer users. Of these eight participants, five were males, seven were current smokers, one was a former smoker, and all used non-menthol tobacco sticks. During the telephone interviews, it appeared that for all users the taste or flavor of the aerosol produced by the vaporizer was the main determinant of the intention to use or to stop using the tobacco vaporizer. The taste or flavor was described as a bad tobacco taste (*n* = 3, including the two former vaporizer users), a burnt taste (*n* = 1), a straw taste (*n* = 1), a tea taste (*n* = 1), a popcorn taste (*n* = 1) and a good tobacco taste (*n* = 1).

A few days after the interviews, these five participants were invited to answer and comment on a preliminary version of the closed format questionnaire, derived from the interviews and from our previous surveys [33]. This phase enabled us to modify and rewrite many questions.

#### Quantitative phase

We enrolled 170 participants, including: 104 users of tobacco vaporizers (Brand 1, *n* = 102; Brand 2, *n* = 1; Brand 3, *n* = 1); 46 incomplete results (respondents who did not mention which product they used); 18 e-cigarette users; one user of nicotine inhaler and one duplicate record.

For homogeneity, and as there were only two users of other types of tobacco vaporizers, we only included the 102 users of the Brand 1 vaporizer in our analysis.

The median age of the 102 participants was 41 years (25th and 75th percentiles: 30 and 51 years; range: 20 to 70 years). About half were women (53%) and current smokers (57% of all respondents; 56% of current users of Brand 1). The distribution of respondents by country was: Switzerland (83%), France (11%), Greece (1%), Italy (1%), Russia (1%), Norway (1%) and Canada (1%).

The majority (76%) of participants scored positively for hazardous alcohol consumption according to the screening test AUDIT-C.

A minority (14%) had used cannabis during the previous 12 months, and 4% had used cannabis at least four times a week during the previous year.

Before they started to use the tobacco vaporizer, all participants were smokers (daily 92%; occasionally 4%) or former smokers (4%). Most current smokers (80%) reported currently trying to reduce their cigarette consumption. Around one third (29%) were trying to quit smoking, but few (9%) had decided to stop smoking, either immediately or within the next 30 days, and a minority (15%) were “very confident” that they could successfully stop smoking if they tried.

Most respondents (96%) were current tobacco vaporizer users; only four (4%) were former vaporizer users. Around one third (35%) were currently also using an e-cigarette, either occasionally (9%) or every day (26%). A minority (7%) were currently also using a nicotine medication either occasionally (2%) or every day (5%). Around one quarter of respondents (27%) were using only the tobacco vaporizer, without concomitant consumption of cigarettes, e-cigarettes or nicotine medications.

The characteristics of these 102 tobacco vaporizer users are summarized in Tables 1 and 2.

**Table 1:** Characteristics of users of the Brand 1 tobacco vaporizer: Internet survey, 2016–2018

	All users
Number of respondents	102
Gender (males %)	47.0
Age (years)^a^	41 (30, 51)
Hazardous alcohol consumption:	
AUDIT-C Score ≥ 4 among males (%)	75.6
AUDIT-C Score ≥ 3 among females (%)	76.1
Ever used cannabis in past 12 months:	
- Ever (%)	14.0
Does your spouse/fiancé smoke	
- Yes (%)	43.0
In general, would you say your health is:	
- Very good to excellent (%)	49.5
Do you currently use a nicotine medication? (patch, chewing-gum, tablet, inhaler, nasal spray)?	
- Yes, every day (%)	5.1
- Yes, occasionally (%)	2.0
Do you currently use an e-cigarette?	
- Yes, every day (%)	26.0
- Yes, occasionally (%)	9.0
Use only vaporizer (no consumption of cigarettes, e-cigarettes or nicotine medication) (%)	26.5

^a^ Median (25th and 75th centiles)

**Table 2:** Characteristics of users of the Brand 1 tobacco vaporizer, Tobacco use: Internet survey, 2016–2018

	All users
Number of respondents	102
Tobacco use: (in current users of the tobacco vaporizer, *n* = 98)	
- Daily smoker (%)	34.8
- Occasional smoker (%)	21.3
- Former smoker (%)	43.8
- Never smoker (%)	0.0
Currently, do you use oral or snuff tobacco:	
- Yes, (occasionally or every day) (%)	8.2
Before using a tobacco vaporizer, were you a smoker (or user of snuff/oral tobacco):	
- Daily smoker (%)	92.0
- Occasional smoker (%)	4.0
- Former smoker (%)	4.0
- Never smoked (%)	0.0
Number of cigarettes per day before using the tobacco vaporizer^a^	20.0 (10.5, 21.5)
The first time you used nicotine, which product did you use:	
- A cigarette, cigar or pipe (%)	98.0
- An electronic cigarette (%)	2.0
Age when began to smoke everyday (years)^a^	16.5 (15.75, 18.0)
Tobacco vaporizer utilization:	
- Every day (%)	88.2
- Occasionally (%)	7.9
- Former user (%)	3.9
Do you intend to use a tobacco vaporizer in the future?	
(Intend to use/certain to use one) (%)	90.3
Among former users: duration of utilization (days)^a^	5.0 (3.0, 5.0)
Current smokers (*n* = 58):	
- Number of cigarettes per day^a^	10.0 (3.75, 16.25)
- How long after waking do you smoke the first cigarette of the day (minutes)^a^	30.0 (10.0, 50.0)
- Heaviness of smoking index (HSI) (%):	
0–1:	31.6
2–4:	68.4
5–6:	0.0
- Cigarette dependency (self-rating scale of 0 to 100)^a^	80.0 (43.75, 96.0)
- Decided to stop smoking (now or in the next 30 days) (%)	9.0
- Intend to stop in the next six months (%)	7.1
- Sure to succeed stopping smoking if you try (very sure) (%)	14.5
- Currently trying to stop smoking (%)	28.6
- Currently trying to reduce cigarette consumption (%)	80.0
- Duration of most recent quit attempt (days)^a^	14 (3.0, 90.0)
- Duration of longest quit attempt (days)^a^	135.0 (21.0, 365.0)
Former smokers (*n* = 40):	
- When did you stop smoking (days ago)^a^	67 (36.0, 438.0)
- Before stopping, number of cigarettes per day on average^a^	20.0 (10.5, 23.0)

^a^ Median (25th and 75th centiles)

#### Tobacco vaporizer utilization

Among the 98 current users of the Brand 1 tobacco vaporizer, the median duration of utilization was 8.8 months (269 days, 25th and 75th percentiles: 59 and 469 days). The majority (94%) used it during both week days and at the weekend; 92% used the vaporizer every day and 8% occasionally (Table 3). Among daily vaporizer users 29% were also smoking every day, whereas among occasional vaporizer users 75% were smoking every day.

**Table 3:** Modes of utilization in current users of the Brand 1 tobacco vaporizer: Internet survey, 2016–2018

	Current users
Number of current users	98
Duration of utilization (days)^a^	269.0 (59.0, 469.0)
Utilization:	
Every day (%)	91.8
Occasionally (%)	8.2
Before using your vaporizer, how many cigarettes did you smoke per day, on average (current daily or occasional vaporizer users who were smokers when they started to use the vaporizer, *n* = 94)^a^	20.0 (10.0, 20.0)
Number of cigarettes per day now (among all current vaporizer users and current smokers)^a^	8.0 (3.0, 15.0)
Number of cigarettes per day now (among every daily vaporizer users and current smokers)^a^	7.5 (3.25, 13.75)
Number of refill sticks per day^a^	10.0 (5.75, 10.0)
Total number of cigarettes plus vaporizer refills per day (in current smokers)^a^	16.5 (12.0, 21.0)
First utilization of the day of vaporizer, how many minutes after waking (minutes)^a^	30.0 (15.0, 60.0)
Number of puffs per day on the vaporizer^a^	150.0 (50.0, 210.0)
Number of puffs per stick^a^	12.0 (10.0, 14.0)
Duration of one recharge of the battery (hours)^a^	24.0 (12.0, 48.0)
Number of sticks per one recharge of the battery^a^	20.0 (20.0, 20.0)
Monthly expenditure for vaporizer and sticks (Euros)^a^	110.0 (59.0, 161.0)
Intention to use vaporizer for more than one year (%)	58.9
Utilization of vaporizer and cigarettes on the same day:	
- Ever (%)	59.2
- For more than one month (%)	15.4
Reason for using both vaporizer and cigarettes on the same day:	
- To reduce cigarette consumption (%)	57.6
- In places where smoking is prohibited (%)	19.7
- Because I like to use both (%)	28.8
Utilization of the vaporizer instead of a smoking cessation medication:	
- Ever (%)	48.1
- For more than one month (%)	28.4

^a^ Median (25th and 75th centiles)

The first puff of the day on the vaporizer took place 30 min (median) after waking (25th and 75th percentiles, 15 and 60 min), and the first cigarette of the day was also smoked 30 min (median) after waking among current smokers (25th and 75th percentiles: 10 and 50 min). Among dual users (current users of both vaporizers and cigarettes), 14.8% took their first puff of the day on their vaporizer within five minutes after waking, and 15.7% smoked their first cigarette of the day within five minutes after waking.

Among the 98 current vaporizer users, the median number of refill sticks per day was ten (25th and 75th percentiles: 5.75 and 10); the median number of puffs per day was 150 (25th and 75th percentiles: 50 and 210) and the median number of puffs per stick was 12 (25th and 75th percentiles: 10 and 14). The median monthly expenditure on tobacco vaporizers and refill sticks was 110 Euros (25th and 75th percentiles: 59 and 161). About half the participants (48%) said they had ever used the tobacco vaporizer instead of a smoking cessation medication to reduce or to quit smoking.

Most users (59%) had ever used the vaporizer and combustible cigarettes concomitantly (i.e. on the same day), and 15% had done so for more than one month. The most frequent reason for concomitant use of both the vaporizer and combustible cigarettes on the same day was to reduce cigarette consumption (58%; other reasons are listed in Table 3).

The majority (84%) of participants intended to continue using the tobacco vaporizer in the future and, among active users, 59% intended to use it for more than one year.

#### Reasons for use

The tobacco vaporizer was used mainly (in decreasing order of frequency) to replace cigarettes; because it was perceived to be less toxic than smoking tobacco; to stop smoking or to avoid starting smoking again; to reduce tobacco consumption with no intention of stopping smoking; and because respondents did not want to smell of tobacco smoke. Other reasons are listed in Table 4.

**Table 4:** Reasons for using the tobacco vaporizer: Internet survey, 2016–2018

Quite true to totally true (%)	All users
Number of respondents	102
To replace cigarettes	94.1 (89.5–98.7)
Less toxic than smoking tobacco	89.2 (83.2–95.2)
To stop smoking or to avoid starting smoking again	72.3 (63.6–81.0)
To reduce my tobacco consumption but without the intention of stopping smoking	71.3 (62.5–80.1)
Because I don’t want to smell of tobacco smoke	70.7 (61.8–79.6)
To cope with tobacco withdrawal symptoms	69.6 (60.7–78.5)
Because I like using it	65.0 (55.7–74.3)
To not disturb others with tobacco smoke	57.4 (47.8–67.0)
To reduce my tobacco consumption in preparation for stopping smoking	56.4 (46.7–66.1)
Because I’m dependent on my vaporizer	53.5 (43.7–63.3)
To manage urges to smoke	49.0 (39.3–58.7)
Because all the other smoking cessation methods I tried have failed	41.6 (32.0–51.2)
Because, despite my efforts, I’m not able to stop using my vaporizer	37.4 (27.9–46.9)
To manage stress	35.3 (26.1–44.5)
To avoid the need to go outside to smoke	26.0 (17.4–34.6)
In situations or places where smoking is prohibited	23.0 (14.8–31.2)
Cheaper than tobacco	14.0 (7.2–20.8)
The vaporizer helps me control my weight	4.0 (0.2–7.8)
I cannot smoke because of a disease	2.0 (0.0–4.7)

95% confidence intervals are indicated in brackets

#### Perceived effects

The majority (59%) of respondents rated the taste of the vaporizer as either good or very good, whereas 6% rated it bad or very bad. About half (45%) described the vaporizer taste as a good tobacco taste, 19% as a straw taste, 19% as a tea taste, 10% as a popcorn taste, 4% as a bad tobacco taste and 3% as a burnt taste. Two thirds (68%) of participants reported that the taste of the vaporizer was either different or very different from the taste of a cigarette. Most (61%) reported that the vaporizer taste to be advantageous for stopping smoking (Table 5).

**Table 5:** Perceived effects of using the Brand 1 tobacco vaporizer: Internet survey, 2016–2018

	All users
Number of respondents	102
How would you describe the ‘hit’ or ‘throat hit’ provoked by your vaporizer:	
- Strong to very strong (%)	14.7 (7.9–21.5)
- Medium (%)	30.4 (21.5–39.3)
- Weak to very weak (%)	54.9 (45.2–64.6)
Would you describe the vaporizer taste as:	
- Good to very good (%)	59.4 (49.8–69.0)
- Neutral (%)	34.7 (25.4–44.0)
- Bad to very bad (%)	5.9 (1.3–10.5)
Is the taste of your vaporizer similar of the taste of a cigarette?	
- Similar (%)	1.0 (0.0–2.9)
- Near to very near (%)	31.0 (22.0–40.0)
- Different to very different (%)	68.0 (58.9–77.1)
Would you say the taste of your vaporizer is an advantage or a disadvantage for stopping smoking?	
- Advantage to big advantage (%)	60.8 (51.3–70.3)
- Disadvantage to big disadvantage (%)	10.8 (4.8–16.8)
Could the taste of your vaporizer make you want to smoke a cigarette?	
- A lot (%)	2.9 (0.0–6.2)
- Low to moderate (%)	28.4 (19.6–37.2)
- Not at all (%)	68.6 (59.6–77.6)
Does your vaporizer irritate your throat?	
- Strongly (%)	9.0 (3.4–14.6)
- Not at all (%)	65.0 (55.7–74.3)
How would you estimate the general risk to health from the vaporizer, compared to cigarettes?	
- 100 times less dangerous (%)	20.8 (12.9–28.7)
- 10 times less dangerous (%)	49.5 (39.7–59.3)
- 2 times less dangerous (%)	21.8 (13.9–29.8)
- Probably the same risk as cigarettes (%)	6.9 (2.0–11.8)
- Probably more dangerous than cigarettes (%)	1.0 (0.0–2.9)
Former smokers (n = 40):	
Did your vaporizer help you to stop smoking?	
- Yes (a little to absolutely) (% of former smokers)	67.5 (53.0–82.0)
Current smokers (n = 58):	
Does (did) your vaporizer help you to reduce your cigarettes consumption?	
- Yes (a little to absolutely) (% of current smokers)	84.5 (75.2–93.8)

95% confidence intervals are indicated in brackets

Among former smokers, 68% answered that the tobacco vaporizer had helped them to stop smoking and, among current smokers, 85% said that the vaporizer helped them to reduce their cigarette consumption.

Current smokers who were daily or occasional vaporizer users reported smoking a median of 8.0 cigarettes per day, compared with 20.0 per day before they started to use the vaporizer (*p* < .0001, Wilcoxon signed-rank test).

Most (92%) participants estimated the vaporizer use to be less dangerous for their health than cigarette smoking: 100 times less dangerous (21%), 10 times less dangerous (49%), two times less dangerous (22%).

A minority (9%) reported strong throat irritation with the vaporizer, and 55% rated the throat hit provoked by the vaporizer as weak or very weak. One third (31%) reported the vaporizer taste could make them want to smoke a cigarette versus 69% who said the vaporizer taste did not make them want to smoke.

#### Satisfaction

On a scale from zero to ten, the median satisfaction score was eight (25th and 75th percentiles: 7 and 9). More than half the users (56%) had ever recommended several other people to use the tobacco vaporizer and 22% thought that several people had begun to use the vaporizer because of their recommendation or because of their example. The nicotine intake from the vaporizer was considered to be sufficient by 90% of users. The majority of ex-smokers (67%) expressed fear that they would start smoking again if they stopped using the vaporizer.

The perceived advantages of the vaporizer were, in decreasing order of frequency: it was easy to not smoke when using the vaporizer; the vaporizer did not produce a bad smell; since starting to use the vaporizer respondents were coughing less; users had better breath; they were less short of breath after a physical effort, and their senses of taste and smell improved (Table 6).

**Table 6:** Satisfaction with, and perceived advantages and disadvantages of, the tobacco vaporizer: Internet survey, 2016–2018

	All users
Number of respondents	102
Are you satisfied with your vaporizer (scale of 0 to 10)^a^	8.0 (7.0, 9.0)
I like the feeling I get when I inhale the vapor from my vaporizer	
- Somewhat agree to totally agree (%)	66.7 (57.4–76.0)
Have you ever recommended other people to use a vaporizer:	
- Yes, one person (%)	24.5 (16.2–32.8)
- Yes, several people (%)	55.9 (46.3–65.5)
Do you think that other people began to use a vaporizer because of your recommendation or your example?	
- Yes, one person (%)	25.7 (17.2–34.2)
- Yes, several people (%)	21.8 (13.8–29.8)
I’m afraid I may start smoking again when I stop using my vaporizer	
- Somewhat agree to totally agree (% of former smokers)	64.7 (56.3–74.1)
Perceived advantages - Somewhat agree to totally agree (%):	
It’s easy not to smoke when I use my vaporizer	82.0 (74.5–89.5)
It does not produce a bad smell	73.0 (64.3–81.7)
I cough less	68.0 (58.9–77.1)
I have better breath	66.0 (56.7–75.3)
I get less short of breath after a physical effort	60.7 (51.1–70.3)
Improved senses of taste and smell	43.0 (33.3–52.7)
Perceived disadvantages: - Somewhat agree to totally agree (%)	
I’m afraid of becoming dependent on my vaporizer	57.6 (47.9–67.3)
The vapor should be more concentrated	24.0 (15.6–32.4)
My vaporizer should act more quickly (faster relief of urge to smoke)	20.2 (12.3–28.1)
It should be easier to draw/inhale on the vaporizer	19.0 (11.3–26.7)
Vaporizer should deliver more nicotine	10.0 (4.1–15.9)

^a^ Median (25th and 75th centiles); other results are proportions with 95% confidence intervals

The perceived disadvantages were, in decreasing order of frequency: users were afraid they would become dependent on the vaporizer; the vapor from the vaporizer should be more concentrated; the vaporizer should act more quickly; it should be easier to inhale on the vaporizer; the vaporizer should provide more nicotine. Side effects of the vaporizer, reported by some users, were: sore throat (*n* = 5), stomach pain (*n* = 4), headache (*n* = 3), dry mouth (n = 3), cough (n = 3), and bad breath (*n* = 2).

In an open-ended question (free text, 26 answers collected) on how the vaporizer could be improved, participants answered that it should be less fragile because it breaks easily (*n* = 4), it should be possible to reduce the nicotine level in the refills (n = 4), there should be a wider choice of tastes (*n* = 3), and various other responses (*n* = 15).

#### Comparison between current and former smokers

We compared current and former smokers for each variable and report here results with a *p* value less than 0.05, although they are not statistically significant when using Bonferroni correction (corrected *p* = 0.0004): former smokers said more frequently than current smokers that their reason for using the vaporizer was that, despite their efforts, they were not able to stop using it (55% versus 26%, *p* = 0.006); that they used it because they were dependent on the vaporizer (72% versus 37%, *p* = 0.004); that they liked using it (74% versus 59%, *p* = 0.025); that they used it to avoid disturbing others with tobacco smoke (70% versus 49%, *p* = 0.04) and because it was less toxic than smoking tobacco (100% versus 81%, *p* = 0.014).

In addition, more former smokers than current smokers responded that the vaporizer taste did not make them want to smoke a cigarette at all (82% versus 59%, *p* = 0.031).

## Discussion

The main findings of this online survey in mostly Swiss and French visitors to an anti-addiction website were that Brand 1 was by far the most frequently used tobacco vaporizer, that the tobacco vaporizer was used mainly to replace cigarettes and that it scored highly in terms of satisfaction. Also, users considered the vaporizer to be less toxic than cigarette smoke, although we used ad hoc (i.e. not formally validated) question to assess this. The vaporizer was perceived to be helpful for reducing cigarette consumption or for stopping smoking, and also to diminish respiratory symptoms such as coughing and shortness of breath after physical effort. All these results should of course be confirmed by experimental studies.

In this online sample, the tobacco vaporizer was used exclusively by current and former smokers. Most current smokers (dual users) reported currently trying to reduce their cigarette consumption and around one third were trying to quit smoking. But only around 10% had decided to stop smoking immediately or in the next 30 days, and their confidence in their ability to successfully quit smoking was low.

Most vaporizer users were also current smokers, but concomitant cigarette smoking reduces the potential of vaporizers to lower the risk of tobacco-related harm.

Among dual users (current users of vaporizers and cigarettes), the proportion of users who smoked their first cigarette within five minutes after waking and the proportion of users who used the vaporizer within five minutes after waking were the same. Time to the first puff is a useful indicator of dependence [29], and this result suggests that the addictiveness of both products is similar.

The side effects reported with the vaporizer (sore throat, stomach pain, headache, dry mouth, cough and bad breath) were rare, but many users feared becoming dependent on the vaporizer.

With respect to product design, the majority of users perceived that the aerosol produced by the tobacco vaporizer was concentrated enough and quickly relieved the urge to smoke [2, 11]. Most participants in our study said it was easy to draw on the vaporizer and that it provided enough nicotine. One crucial point that may explain the success of the Brand 1 vaporizer - that could also explain the failure of first generation heat-not-burn tobacco products, because they scored poorly in this respect [34] - is that the satisfaction produced with Brand 1 tobacco vaporizer seems to be due to its taste, which was well liked, while the “throat hit” was described as only medium to weak. The majority of vaporizer users described the taste as good, different from the taste of a cigarette and helpful for stopping smoking, and the majority said the taste would not make them want to smoke a cigarette.

According to users, the vaporizer could be improved by providing refills with lower nicotine content to allow users to reduce their nicotine intake, by making the device less fragile and by expanding the choice of tastes.

Participants in our survey had relatively high rates of hazardous alcohol consumption (positive AUDIT-C test), which is in accordance with the high rates of hazardous alcohol consumption in smokers [35, 36] and is probably not specific to Brand 1 tobacco vaporizer users. Participants also had a high rate of cannabis consumption, which is in accordance with the high rate of cannabis consumption usually observed in smokers [36] and is probably not specific to Brand 1 vaporizer users. Nevertheless, these high levels of alcohol and cannabis use must be kept in mind when considering tobacco vaporizer users.

### Study strengths and limitations

Although a new systematic review that compared industry-funded with independent studies of heated tobacco products [20] found that independent and industry-funded studies produced largely similar findings, our study is innovative and the aspects covered have not been previously reported by independent researchers who are not linked to the manufacturers of tobacco vaporizers.

We used a practical and feasible way of enrolling tobacco vaporizer users, and had to rely on an online survey in self-selected volunteers. We therefore had no way of ensuring that the respondents to our online questionnaire were actually using the brand of tobacco vaporizer that they mentioned. Participants were among the first users of this product, soon after it was launched, and are innovators and early adopters that may differ from the late majority [37]. In the qualitative phase, the sample size (*n* = 8) may have been too small to reach data saturation, and a larger sample may have provided more information. In the quantitative phase, the number of participants was lower than intended, probably owing to the novelty of tobacco vaporizers and, given the very low prevalence of heated tobacco product use at the time of data collection, obtaining a representative sample of 200 users would have required a prohibitively large survey, which was not feasible given our resources. For these reasons, our study only included a small sample of innovators and early adopters mainly from Switzerland and France and may not be representative of all Brand 1 vaporizer users in all countries. Moreover, participants were recruited via an anti-addiction website and may have been more motivated to reduce or stop smoking than other Brand 1 vaporizer users. Users who take part in online survey research may also differ from other vaporizer users, in that they may be more educated. Thus, participants in our study may differ from average Brand 1 vaporizer users, and our results may have limited generalizability. Finally, we used an ad hoc questionnaire that had not been submitted to formal validation, although it was pre-tested online and iteratively improved in eight participants. The questionnaire for the quantitative phase was designed for users of all types of tobacco vaporizers, but because all participants in the qualitative phase were using Brand 1, we may have missed some elements specific to other types of vaporizers when designing the questionnaire. It should be noted that some of the other currently available vaporizers were not yet on the market when the questionnaire was designed.

Nonetheless, despite its limitations, this exploratory study contributes valuable information about who uses tobacco vaporizers and how and why such products are used. Further research should be conducted in more representative samples of tobacco vaporizer users, include other brands of tobacco vaporizers, and use experimental methods.

## Conclusions

In this online, self-selected sample of early adopters, the Brand 1 tobacco vaporizer was by far the most frequently used tobacco vaporizer. It was used by current or former smokers only, mainly to replace cigarettes, and satisfaction ratings were good. Users considered the tobacco vaporizer to be less toxic than cigarette smoke and perceived it to be helpful for reducing or stopping smoking.

## Abbreviations

AUDIT-C: Alcohol Use Disorders Identification Test-Consumption
GBP: Great Britain Pound
HSI: Heaviness of Smoking Index
IQOS: I Quit Ordinary Smoking
PMI: Philip Morris International
